# Patient Perceptions of a Personal Health Record: A Test of the Diffusion of Innovation Model

**DOI:** 10.2196/jmir.2278

**Published:** 2012-11-05

**Authors:** Srinivas Emani, Cyrus K Yamin, Ellen Peters, Andrew S Karson, Stuart R Lipsitz, Jonathan S Wald, Deborah H Williams, David W Bates

**Affiliations:** ^1^Division of General Internal Medicine, Department of MedicineBrigham and Women's HospitalHarvard Medical SchoolBoston, MAUnited States; ^2^Department of Emergency MedicineCollege of MedicineUniversity of CincinnatiCincinnati, OHUnited States; ^3^Department of PsychologyThe Ohio State UniversityColumbus, OHUnited States; ^4^Clinical Decision Support Unit, Department of MedicineMassachusetts General HospitalHarvard Medical SchoolBoston, MAUnited States; ^5^RTI International, Waltham, MA, United StatesHarvard Medical SchoolBoston, MAUnited States; ^6^Department of Healthcare Policy and ManagementHarvard School of Public HealthBoston, MAUnited States

**Keywords:** Personal health record (PHR), perceptions, innovation, electronic health records (EHRs), meaningful use

## Abstract

**Background:**

Personal health records (PHRs) have emerged as an important tool with which patients can electronically communicate with their doctors and doctor’s offices. However, there is a lack of theoretical and empirical research on how patients perceive the PHR and the differences in perceptions between users and non-users of the PHR.

**Objective:**

To apply a theoretical model, the diffusion of innovation model, to the study of PHRs and conduct an exploratory empirical study on the applicability of the model to the study of perceptions of PHRs. A secondary objective was to assess whether perceptions of PHRs predict the perceived value of the PHR for communicating with the doctor’s office.

**Methods:**

We first developed a survey capturing perceptions of PHR use and other factors such as sociodemographic characteristics, access and use of technology, perceived innovativeness in the domain of information technology, and perceptions of privacy and security. We then conducted a cross-sectional survey (N = 1500). Patients were grouped into five groups of 300: PHR users (innovators, other users, and laggards), rejecters, and non-adopters. We applied univariate statistical analysis (Pearson chi-square and one-way ANOVA) to assess differences among groups and used multivariate statistical techniques (factor analysis and multiple regression analysis) to assess the presence of factors identified by the diffusion of innovation model and the predictors of our dependent variable (value of PHR for communicating with the doctor’s office).

**Results:**

Of the 1500 surveys, 760 surveys were returned for an overall response rate of 51%. Computer use among non-adopters (75%) was lower than that among PHR users (99%) and rejecters (92%) (*P* < .001). Non-adopters also reported a lower score on personal innovativeness in information technology (mean = 2.8) compared to 3.6 and 3.1, respectively, for users and rejecters (*P* < .001). Four factors identified by the diffusion of innovation model emerged in the factor analysis: ease of use, relative advantage, observability, and trialability. PHR users perceived greater ease of use and relative advantage of the PHR than rejecters and non-adopters (*P*<.001). Multiple regression analysis showed the following factors as significant positive predictors of the value of PHR for communicating with the doctor’s office: relative advantage, ease of use, trialability, perceptions of privacy and security, age, and computer use.

**Conclusion:**

Our study found that the diffusion of innovation model fits the study of perceptions of the PHR and provides a suitable theoretical and empirical framework to identify the factors that distinguish PHR users from non-users. The ease of use and relative advantage offered by the PHR emerged as the most important domains among perceptions of PHR use and in predicting the value of the PHR. Efforts to improve uptake and use of PHRs should focus on strategies that enhance the ease of use of PHRs and that highlight the relative advantages of PHRs.

## Introduction

In the last decade, electronic personal health records (PHRs) have emerged as an important Internet-based tool with which patients can communicate with their doctor’s offices for tasks such as accessing components of the electronic health record (EHR), requesting appointments and prescription refills, and asking non-urgent medical questions. The Markle Foundation’s Connecting for Health was a landmark report in recognizing the potential value of PHRs [1]. Since then, a number of reports and reviews have also discussed the importance of PHRs in helping patients take a more active role in their health care [2-6]. The regulations associated with meaningful use of electronic health records (EHRs), developed as a result of the Health Information Technology for Economic and Clinical Health (HITECH) Act [7], have underscored the importance of PHRs in the United States, and they include several core and menu measures for the electronic exchange of information between providers and patients that are likely to substantially increase adoption rates of these tools. In addition, a number of empirical studies have focused on adoption and use rates and patient satisfaction with PHRs [8-20].

Collectively, the empirical studies and reviews of PHR adoption and use offer some insights into the experience with PHRs over the last decade. The first is the emergence of two basic types of PHRs categorized as tethered (to the EHR of the provider) and untethered (or standalone) [2,6]. There has been a steady increase in implementation and uptake of tethered PHRs in the United States. For example, Kaelber and colleagues [3] reported that about fifty million patients have access to the MyChart PHR tethered to the Epic EHR system, and another twenty million veterans have access to My Health*e*Vet, the Department of Veterans Affairs PHR. Standalone PHRs are either created and maintained by the individual on a personal computer or accessible as Internet-based applications such as Microsoft’s HealthVault. Internet-based standalone PHRs have been less successful in terms of uptake compared to tethered PHRs, and one prominent standalone PHR, Google Health, was discontinued in January 2012 because of lack of widespread adoption.

As the uptake and use of tethered PHRs grew, empirical research emerged on adoption and use rates of and satisfaction with PHRs. In terms of PHR use, the most commonly used functionalities of PHRs are review of medical test results (laboratory and radiology results), requests for medication refills, and clinical messaging with the provider and practice [9,11,12,14,15,18,20]. Among sociodemographic factors influencing use, race (being Caucasian) and income (high socioeconomic status) have been found to be significant predictors of use, suggesting the presence of a digital divide [9,12,17,20]. Patients with high expected need of clinical services and presence of chronic/comorbid conditions are more likely to use the PHR [9,13,14]. An outcome of interest in most empirical studies on use has been patient satisfaction, with patients reporting high levels of satisfaction with PHRs [8,11,14,21]. In terms of clinicians, some studies have reported that clinicians are less likely than patients to anticipate benefits from PHRs and were concerned with the impact on workload, especially unreimbursed work, as a result of PHR use [22,23]. However, clinician workload from PHRs has been found to be lower than anticipated and clinicians are generally satisfied with PHRs [8,11,21,24]. Nonetheless, clinician encouragement and use of PHRs has been found to be an important driver of patient adoption and use of PHRs [18].

The third set of insights related to the PHR experience focuses on issues such as functionality or purpose, system attributes, and privacy and security. Kaelber and colleagues [3] summarized four primary functions of a PHR: (1) Information collection (from the EMR as well as patient-entered data); (2) Information sharing (one-way sharing of information with others); (3) Information exchange (two-way exchange of information); and (4) Information self-management (such as tracking information and decision support). Of these functions, existing PHRs are predominantly aimed toward information exchange and information self-management. The information collection component, specifically patient-entered data in the PHR, lags behind for several reasons including: logistical (eg, workflows around who should review and accept the data into the PHR) and legal (eg, liability if data is not reviewed on a timely basis) [5]. Similarly, patient sharing of PHR information across organizations remains problematic because of interoperability issues. In the case of privacy and security issues associated with PHRs, the role of privacy and security in influencing adoption of PHRs and the need to balance enhanced security of information in PHRs with ease of use of PHRs has been noted [3,6]. However, several studies have reported that once patients adopt and use PHRs, they are less likely to be concerned with privacy and security issues [8,25].

In spite of the progress on research on PHRs, several gaps remain in the current literature. First, our review of PHR research as well as that conducted by others [6] has found an overwhelming emphasis on patient satisfaction as an outcome measure and a corresponding lack of inclusion of other outcome measures. For example, one outcome of interest identified by Archer and colleagues [6] is sustainability or the degree of PHR use after adoption. Our analysis of PHR research has identified the perceived value of the PHR as an outcome measure. Beyond outcomes, there has also been little research conducted in terms of factors such as perceptions of PHRs and their impact on outcomes. For example, if patients perceive a PHR to be easy to use and having an advantage over a traditional approach such as calling the doctor’s office for tasks such as medication refills or appointment requests, then they will likely perceive greater value of the PHR. Thus, there is a need to go beyond satisfaction as an outcome measure and at the same time understand the role of predictors such as perceptions on outcomes. Second, most of the PHR research has focused on adopters, and we know little about characteristics of non-adopters and how non-adopters perceive and value PHRs. Finally, little work has been done to apply theoretical frameworks to the study of patient adoption and use of PHRs. A suitable theoretical framework and associated concepts can not only advance our understanding of why patients adopt and use the PHR but also generate prescriptive findings on improving the adoption and use of this important tool. For example, such prescriptive findings can consist of strategies that organizations can adopt to promote positive perceptions of PHRs in order to influence adoption and use rates.

In this paper, we seek to collectively address these three gaps in the research on PHRs. A PHR represents an innovation for patients as it partially replaces the existing practice of calling the doctor’s office for an appointment request or a prescription refill with an electronic, Internet-based approach to the same tasks. Moreover, as Rogers [26] points out, it is the perception of the innovation that matters since it is perceptions rather than actual attributes that will influence adoption. Tang and colleagues [2] have noted that “widespread adoption and use of PHRs will not occur unless they provide perceptible value to users, are easy to learn and easy to use”. Therefore, in this study we applied a well-known and widely recognized theoretical framework, the diffusion of innovation model [26], in assessing patient perceptions of PHRs. Given the different functions of the PHR identified in the introduction above, the focus of our application of the diffusion of innovation model is to assess perceptions of PHR as a partial replacement for calling the doctor’s office for communication functions such as appointment requests, medication refills, or asking the doctor a non-urgent medical question.

## Methods

### The Diffusion of Innovation Model

After a review of hundreds of innovation studies spanning fields such as agriculture, information technology, and pharmaceuticals, Rogers [26] identified five perceived attributes in the diffusion of innovation model that are most likely to influence the adoption of an innovation: (1) Relative Advantage, or the degree to which an innovation (such as a PHR) is perceived as being better than the idea it supersedes (for example, calling the doctor’s office); (2) Compatibility, or the degree to which an innovation is perceived as consistent with existent values, past experiences, and needs of potential adopters (as, for example, past experience with using the Internet); (3) Complexity, or the degree to which an innovation is perceived as easy to understand and used as a whole or in incremental parts; (4) Trialability, or the degree to which an innovation can be experimented with on a limited basis (for example, trying a PHR for tasks such as appointment requests or secure messaging); and (5) Observability, or the degree to which the benefits of an innovation are visible to intended adopters. Each of these five perceived attributes is positively related to the rate of adoption of an innovation as well as its use.

A comprehensive approach to empirical research in this area was undertaken by Moore and Benbasat [27] who proposed that the focus of research should be on perceptions of innovation *use* rather than perceptions of innovation attributes since behavioral intentions are best explained by use perceptions (following Fishbein and Ajzen’s theory of the relationship between attitudes and behaviors) [28]. As a result, Moore and Benbasat developed a survey to measure perceptions of innovation use. Our study followed the approach of Moore and Benbasat to focus on perceptions of the use of the PHR. We selected and modified a set of survey items developed by Moore and Benbasat concerning perceptions of use of a personal work station (PWS) to fit perceptions of using a PHR. For example, one of the items in the domain of Relative Advantage developed by Moore and Benbasat was “Using a PWS gives me greater control over my work”. We modified the wording of this item to fit our study as follows: “Using a PHR gives me greater control over my care”. Our modification of the wording also reflected our focus on non-users of a PHR in this study. For non-users, our survey item for the previous example captured potential use of a PHR: “Using a PHR will give me greater control over my care”. We developed items for the five domains of perceptions in the diffusion of innovation model identified by Rogers: relative advantage, compatibility, ease of use (or complexity), trialability, and observability.

Beyond perceptions of innovation use, other research on adoption and use of innovations has focused on factors that may modify perceptions of innovations. One such set of factors, personal innovativeness in the domain of information technology (PIIT), was developed by Agarwal and Prasad [29]. PIIT is defined as “the willingness of an individual to try out new information technology” [29] and captures individual-level differences in the innovation-decision model. PIIT may play a particularly important role in distinguishing PHR users from non-users; for example, do PHR users have greater levels of PIIT than non-users? Moreover, such individual-trait variables have not been examined in existing research on PHRs, which has focused for the most part on sociodemographic characteristics [9,11-17]. For our study, we selected, without any modifications, four survey items capturing PIIT developed by Agarwal and Prasad [29]. These items were generically worded to fit our study as, for example: “I like to experiment with new information technologies” and “Among my peers I am usually the first to try out new information technologies”. A third set of items included in our study pertains to the privacy and security of information in the PHR as concerns about privacy and security can play a key role in whether patients adopt and use PHRs [3-6]. In addition to these items on perceptions, we included items on sociodemographic characteristics (for example, age, gender, income, education, and race), and technology use and access.

The outcome measure of interest in this study is the perceived value of the PHR for communicating with the doctor’s office. Several studies have pointed to the importance of assessing this outcome measure. As noted above, Tang and colleagues [2] call attention to the “perceptible value” of the PHR for users. They also point to the importance of understanding whether the cost (financial and effort) of adopting a PHR can be justified related to the perceived value of the PHR. In suggesting additional topics of research related to their analysis of eHealth services, Hsu and colleagues [9] recommend a study of perceived need and value of such services compared to alternatives. Finally, Kaelber and colleagues [3] distinguish between measurable value of PHRs such as improved quality and better patient satisfaction and perceived value of PHRs. They argue that perceived value may drive PHR adoption and use even if PHRs do not provide measurable value. Therefore we adopted the perceived value of the PHR for communicating with the doctor’s office as the outcome measure for this study. We measured perceived value of the PHR for communicating with the doctor’s office on a scale from 1 to 10 with 1 indicating no value at all and 10 indicating highly valuable.

To summarize, based on the literature on diffusion of innovations we identified five perceived attributes of innovation use: relative advantage, compatibility, ease of use, trialability, and observability. From the literature on information technology use, we identified a variable, personal innovativeness in information technology, which may help distinguish the level of innovativeness between PHR users and non-users. From the PHR literature, we identified several factors that play a role in the adoption and use of PHRs: perceptions of privacy and security, sociodemographic characteristics, and technology use including computer and Internet use. All these variables comprise our predictor variables. The PHR literature also yielded the outcome measure of the study, perceived value of the PHR in communicating with the doctor’s office. To achieve the objective of our study, we will empirically test the application of the diffusion of innovation model to PHRs. That is, we will test for the presence of the five perceived attributes of PHR use. We will then assess the significance of the various predictors including the perceived attributes of innovation use in predicting our outcome measure. We will also conduct analysis to compare users and non-users with respect to the different predictors and the outcome measure.

Our approach is a cross-sectional survey of users and non-users of the PHR. First we developed a draft survey based on relevant items and findings from the existing literature as discussed above. Once the survey was developed, we conducted an internal test of the survey using several staff, some of whom were users while others were non-users of a PHR. Based on this testing, we eliminated some items and revised others. For example, we adopted some reverse-worded items to minimize response bias in the survey. We developed two versions of our survey: (1) a user version in which we asked about patient perceptions of using a PHR; and (2) a non-user version aimed at non-users of the PHR in which we asked about perceptions of potential use of the PHR. Appendix 1 shows the set of items in the user version of the survey.

### Recruitment

To select the patients for the study, we relied on the definitions of adopter groups in the diffusion of innovation model. Rogers [26] identified five adopter groups in his diffusion of innovation model with respect to their time of adoption: innovators (usually first to adopt an innovation), early adopters, late adopters, early majority, and laggards (generally last to adopt an innovation). For this study, we combined the middle three groups (early adopters, late adopters, and the early majority) into one group called other users and divided patients who used the PHR into three groups: innovators, laggards, and other users. We defined innovators as patients who enrolled (signed up) for the PHR and used it at least once (eg, for a medication refill request or secure messaging with their provider) in a ninety-day (three-month) period after their practice went live with the PHR. Thus innovators were among the first group of patients to adopt the PHR. By specifying that they used the PHR at least once during the ninety-day period, we also ensured that innovators are users. Laggards were defined as adopters who enrolled for the PHR one year after their practice went live with the PHR and then waited for one more year to use the PHR. Thus our definition of laggards considers the lag in time in both adoption and use. Other users were all patients other than innovators and laggards who used the PHR at least once. We realize that these are heuristic definitions of three types of adopter groups based on the diffusion of innovation model. In this initial evaluation, our approach was to develop and apply a simple set of definitions of the adopter groups. We believe that the groups we created allow an initial test of the diffusion of innovation model in PHR research. The three groups of patients—innovators, laggards, and other users—comprise PHR users in our study. Rogers [26] does not consider non-users in his categories of innovators, but we identified two groups of non-users in our research. The first group, which has been the focus of most PHR research, is the non-adopter group comprising patients who never adopted the PHR. The second group of patients is the rejectergroup, ie, patients who adopted the PHR but never used it (these are patients who signed up for the PHR but never used their account even once after enrolling). Thus, our study population was divided into five groups labeled: innovators, laggards, other users, rejecters, and non-adopters.

The study was implemented in the ambulatory care practices of two academic medical centers (Brigham and Women’s Hospital and Massachusetts General Hospital) at Partners HealthCare, an integrated delivery system (IDS) located in Eastern Massachusetts. Partners developed its own tethered-PHR, Patient Gateway, following its strategy of developing and implementing its own electronic health record, the Longitudinal Medical Record (LMR). The Partners PHR was first implemented in 2002 and, at the time of this study, about 80,000 patients had enrolled in the PHR at the two academic medical centers. The PHR has functionality similar to other tethered PHRs including requests for appointments, prescription refills and referrals, access to certain components of the EHR such as laboratory results, and secure messaging with the practice and provider.

The PHR transactions are stored permanently in the Partners clinical information systems and can be accessed for research purposes after IRB approval. We analyzed transactions such as the PHR account created date and the use of different PHR functionalities to identify patients in four of our five groups: innovators, laggards, other users, and rejecters. In the case of non-adopters, we used our scheduling system to identify patients who had a visit at a practice using the PHR but who did not have a PHR account. We selected a random sample of 300 patients for each group for a total of 1500 patients for the patient survey. In the case of non-adopters, we also specified that the patients must have one of four chronic conditions (asthma, CHF, hypertension, or diabetes), which we identified through the problem list in our EHR system. We selected these chronic conditions for non-adopters to ensure that non-adopters have a perceived need and potential reason for using a PHR to communicate with their doctor’s offices. We adopted Dillman’s tailored design method (TDM) to enhance response rates [30]. We sent an introductory letter informing patients that they would be receiving a survey and allowed them the opportunity to opt out of the survey. After removing patients who refused participation in the study in response to the introductory letter, we sent the survey with a $2 cash incentive. We then sent a reminder postcard followed by a reminder survey. All study materials and methods were approved by the Partners HealthCare Institutional Review Board.

### Statistical Analysis

We present frequencies and means of sociodemographic characteristics, patient characteristics, and factors related to technology access and use for our five patient groups. To assess for differences between the five groups, we conducted chi-square tests for categorical data (Pearson’s chi-square for dichotomous and nominal variables) and robust one-way ANOVA for continuous variables. We also computed post-hoc Bonferroni *P*values. We used factor analysis to identify the factor structure of the items pertaining to perceptions of PHR use. Given the exploratory nature of our study, our factor analysis was also exploratory and consisted of principal components analysis with varimax rotation and extraction based on eigenvalues greater than 1 and confirmed by scree plot. We reviewed the Kaiser-Meyer-Olkin (KMO) measure of sampling adequacy and Bartlett’s Test of Sphericity to ensure appropriateness of factor analysis for the data [31]. Based on the results of the factor analysis, we created scales for the different factors using an average of the original data for the items comprising each scale. We also employed multiple regression analysis using a forced entry method to assess predictors of our dependent variable, value of PHR for communicating with the doctor’s office. All analyses were conducted using the SPSS 19.0 statistical software package.

## Results

### Response Rates

Of the 1500 surveys, 760 surveys were returned for an overall response rate of 51%. Response rates varied considerably between the PHR user and non-user groups. In the user group, 173 of 300 (57.7%) of innovators, 179 of 300 (59.7%) of other users, and 162 of 300 (54%) of laggards returned the survey. In comparison, in the non-user group, 118 of 300 (39.3%) of rejecters and 116 of 300 (38.7%) of non-adopters returned the survey. Our response rate for users (innovators, other users, and laggards) exceeds that of previous studies on patient satisfaction with PHRs [8,10]. Our response rate for non-users (rejecters and non-adopters) is comparable to the response rates of users in these studies. Non-responders were younger than responders in the case of innovators and laggards, but there were no differences among the other groups. There were no differences between non-responders and responders with respect to age or gender.

### Sociodemographics Characteristics Among Survey Respondents

Table 1 shows data on sociodemographic characteristics among survey respondents. Innovators were younger (mean age = 55 years) than other users (60 years) and non-adopters (62 years) (*P* = .001), but there were no differences in age among the other groups. Women were represented less in the laggard group (52%) than among innovators (79%), rejecters (75%), and non-adopters (72%) (*P* < .001). The percentage of Caucasian non-adopters (76%) was lower than the percentage of Caucasian innovators (94%), laggards (94%), and other users (86%) (*P* < .001). Only 50% of non-adopters had a four-year college degree or more compared to 76% of the innovators, 71% of laggards, and 69% of other users (*P* = .001), and only 41% of non-adopters had a total household income of $75,000 or more compared to 75% of laggards, 72% of innovators, and 63% of other users (*P* < .001). Non-adopters also differed from innovators and laggards on marital status (47% married; *P* < .001). In terms of overall health status, non-adopters reported a lower rating of overall health compared to innovators and laggards, and other users and rejecters reported lower overall health status than innovators. Innovators also reported a smaller number of comorbidities (mean = 2.8) than other users, rejecters, and non-adopters (mean = 3.7).

**Table 1:** Sociodemographics and self-reported health status of survey respondents.

Groups	Age (years)	Gender (% female) (n)	Race (% Caucasian) (n)	Education^a^	Income^b^	Marital status (% married)	Rating of overall health^c^ (mean)	Self-reported comorbi- dities^d^ (mean)
Innovators	55.4	79% (136/173)	94% (163/173)	76% (126/166)	72% (108/150)	68% (117/173)	3.8	2.8
Other users	59.8	65% (116/179)	90% (161/178)	69% (120/173)	63% (96/153)	63% (112/179)	3.5	3.4
Laggards	59.0	52% (84/162)	94% (152/162)	71% (112/157)	75% (103/137)	70% (114/162)	3.6	3.3
Rejecters	58.3	75% (89/118)	86% (101/118)	61% (68/112)	58% (61/105)	64% (76/118)	3.4	3.5
Non-adopters	61.7	72% (84/116)	76% (88/116)	50% (55/109)	41% (41/101)	47% (54/116)	3.2	3.7
*P* value^e^	*P*=.001	*P*<.001	*P*<.001	*P*=.001	*P*=.001	*P*=.001	*P*=.001	*P*=.001

^a^Education is captured as 4-year college graduate or more.

^b^Income is captured as $75,000 or more in total household income from all sources before taxes.

^c^Rating of overall health is captured as: Excellent (5), Very Good (4), Good (3), Fair (2), or Poor (1).

^d^Self-reported comorbidities included 11 conditions such as: allergies, high blood pressure, high cholesterol, diabetes, heart disease, and asthma or emphysema.

^e^ *P* value for comparison of all five groups.

### Access and Use of Technology

We asked patients about their access to technologies such as a DVD player, iPod/MP3 player, and cell phones and smart phones, use of computers and the Internet, and patient perceptions of personal innovativeness in information technology (PIIT; Table 2). We also gathered data on self-reports of use and satisfaction with the PHR among the three PHR user groups. The three user groups (innovators, other users, and laggards) did not differ from each other on technology access, computer use, Internet use, self-reports of PHR use, and satisfaction with and value of PHR for communicating with their doctor’s office (Table 2). As a result, for the remaining analysis, we combined the three groups (innovators, other users, and laggards) into one group called PHR users, leaving us with three groups for the study: users, rejecters, and non-adopters.

With respect to non-adopters, in terms of technology access (such as use of an iPod/MP3 player, and smartphone/ BlackBerry), non-adopters reported access to a smaller number of technologies (mean = 6.1) compared to users (mean = 7.8) and rejecters (mean = 7.0) (*P* < .001). Computer use among non-adopters was also lower with 87 of 116 (75%) reporting that they used a computer compared to 509 of 514 (99%) for users and 109 of 118 (92%) for rejecters (*P* < .001). Internet use was reported by 82 of 116 (71%) of non-adopters compared to 509 of 514 (99%) for users and 106 of 118 (90%) for rejecters (*P* < .001). Non-adopters also reported a mean PIIT score of 2.8 compared to 3.6 and 3.1, respectively, for users and rejecters (*P* < .001). Thus, non-adopters differed systematically from users of PHRs on sociodemographics, use of technology, and personal innovativeness in information technology. Non-adopters also differed from rejecters of a PHR on access and use of technology, PIIT, and the value of PHR for communicating with the doctor’s office. Overall, the differences between non-adopters and users were greater than those between rejecters and users.

**Table 2:** Access and use of technology among survey respondents.

Groups	Technology access^a^ (mean)	Computer use^b^ (% patients) (n)	Internet use^c^ (% patients) (n)	PIIT^d^ (mean)	Value of PHR^e^ (mean)	Mean # of requests via PHR^f^	Mean satisfaction with PHR^g^
Innovators	7.8	99% (172/173)	99% (172/173)	3.7	6.4	10.0	3.6
Other users	7.8	99% (177/179)	99% (177/179)	3.6	6.7	6.7	3.6
Laggards	7.8	99% (160/162)	99% (160/162)	3.4	6.8	8.3	3.4
Users	7.8	99% (509/514)	99% (509/514)	3.6	6.7	4.1	3.5
Rejecters	7.0	92% (109/118)	90% (106/118)	3.1	6.1	—	—
Non-adopters	6.1	75% (87/116)	71% (82/116)	2.8	4.9	—	—
*P* value^h^	*P*<.001	*P*<.001	*P*<.001	*P*<.001	*P*<.001	.23	.26

^a^A factor developed by adding ten items on access to technology such as a VCR, DVD player, Camcorder, iPod/MP3 player, and smartphone/BlackBerry. A score of 0 indicates that the patient did not have access to all 10 technologies, whereas a score of 10 indicates that the patient had all 10 technologies.

^b^Computer use means that patient was able to use a computer at any of the following locations on at least an occasional basis: home, work, school, library, friend’s house, community center, and other.

^c^Internet use means that patient was able to go online to use the Internet from home, work, or elsewhere.

^d^PIIT refers to personal innovativeness in the domain of information technology.

^e^Value of PHR for communicating with the doctor’s office is measured on a scale from 0 to 10, with 0 meaning no value at all and 10 meaning highly valuable.

^f^Requests made via PHR in a twelve-month period for functions such as appointments, referrals, address and telephone corrections, medication refills, and asking questions about care.

^g^Satisfaction with PHR is measured as: 1 = Excellent, 2 = Very Good, 3 = Good, 4 = Fair, 5 = Poor. Item was reverse coded for results reported in the table.

^h^ *P* value for comparison of user, rejecter, and non-adopter groups except for log-on to PHR and mean satisfaction with PHR for which *P*value is comparison of innovators, other users, and laggards.

### Diffusion of Innovation Theory and Perceptions of Using a PHR

Given that little empirical research has been conducted on the application of diffusion theory to adoption and use of PHRs, we employed exploratory factor analysis with orthogonal rotation to identify the factor structure in our data and assess whether the factors that emerged reflect the theoretically specified domains (such as Relative Advantage and Ease of Use). Our initial factor solution yielded four factors. In this solution, however, items from one domain — Compatibility — loaded highly on several factors indicating that the items on this domain mixed with other domains such as Relative Advantage (the discussion section below elaborates on this finding). In addition, an item on ease of use also loaded highly on several factors and an item on trialability loaded on the ease of use factor, suggesting that these two items were either too complex or did not capture their domains as intended. We removed all such items and conducted the exploratory factor analysis again with the remaining items. Table 3 shows the results of this factor analysis. Four factors with eigenvalues greater than 1 emerged accounting for 72% of the variance in the data. Each factor represents a domain in the diffusion of innovation theory: Factor 1, Ease of Use; Factor 2, Relative Advantage; Factor 3, Observability; and Factor 4, Trialability. Ease of Use accounted for 25% of the variance in the data, Relative Advantage for 21%, Observability 14%, and Trialability 12%.

**Table 3:** Factor analysis of perception items (rotated component matrix^a^).

	Component			
	1 (Ease of use)	2 (Relative advantage)	3 (Observability)	4 (Trialability)
Learning to use PHR was easy for me. (EU^b^)	0.83			
Using PHR is frustrating. (EU)	-0.83			
Using PHR requires a lot of mental effort. (EU)	-0.81			
Overall, I believe that PHR is easy to use. (EU)	0.81			
Using PHR improves the quality of care I receive. (RA)		0.84		
Using PHR gives me greater control over my care. (RA)		0.78		
The effectiveness of care I receive will not improve by my using PHR. (RA)		-0.72		
Using PHR enables me to contact my doctor’s office more quickly. (RA)		0.70		
I have seen what others can do using PHR. (OB)			0.91	
I have talked to others about using PHR. (OB)			0.87	
I tried PHR on a trial basis to see what it can do for me. (TA)				0.85
I really did not lose much by trying PHR, even if I would not have liked it. (TA)				0.80
Eigenvalue	4.4	1.6	1.4	1.3
Percent variance	25	21	14	12
Cronbach alpha for scale	0.88	0.85	0.76	0.57
Mean of scale for PHR User, Rejecter, and Non-Adopter groups	User: 4.0 Rejecter: 3.4 Non-adopter: 3.2	User: 3.4 Rejecter: 3.2 Non-adopter: 2.9	User: 2.5 Rejecter: 2.6 Non-adopter: 2.1	User: 3.5 Rejecter: 3.8 Non-adopter: 3.2
*P* value for comparison of scale among patient groups	*P*<.001	*P*<.001	*P*<.001	*P*<.001

^a^Values below 0.40 have been suppressed.

^b^Indicates the domain of diffusion of innovation that the item belongs to: EU = Ease of Use; RA = Relative Advantage; OB = Observability; TA = Trialability

Our next step was to create scales for each of the four domains. Table 3 also shows the results of the reliability analysis (Cronbach alpha) for each scale (as represented by the factor or domain). Cronbach alpha was very good for Ease of Use (0.88; 4 items) and Relative Advantage (0.85; 5 items), satisfactory for Observability (0.76; 2 items), and fair for Trialability (0.57; 2 items). We then created a scale for each domain by averaging the scores of the items for each domain. Next, we conducted analyses to assess whether our patient groups (PHR users, rejecters, and non-adopters) differed on the four domains. PHR users perceived a greater ease of use of the PHR (Mean = 4.0) than rejecters (Mean = 3.4) and non-adopters (Mean = 3.2), but rejecters and non-adopters did not differ from each other. The same finding holds for relative advantage for which PHR users perceived a greater relative advantage of the PHR (Mean = 3.4) than rejecters (Mean = 3.1) and non-adopters (Mean = 2.9), but rejecters and non-adopters did not differ. In the case of Observability, PHR users and rejecters perceived greater observability of the PHR than non-adopters but did not differ from each other. In the case of Trialability, all three groups differed from each other with rejecters reporting greater perceptions of trialability than users and non-adopters.

### Predicting the Value of a PHR for Communicating With the Doctor’s Office

Our final analysis assessed the role of different variables in predicting our dependent variable, patient perceptions of the value of the PHR for communicating with their doctor’s office measured on a scale from 0 = no value at all to 10 = highly valuable. Our independent variables consisted of: sociodemographic variables such as age, gender, education and income, access and use of technology, PIIT, a factor on patient perceptions of privacy and security (created by combining three items on privacy and security; Appendix 1), and the four scales representing the domains of the diffusion of innovation model from Table 3. We also retained our patient groups of PHR users, rejecters, and non-adopters in the analysis. Table 4 shows the results of the multiple regression analysis when all variables are entered into the model.

The R-square for the model fit is 0.51 (*P*<.001). Six items are significant predictors of the value of the PHR for communicating with the doctor’s office. Three of the items are domains of perceptions of innovation use from the diffusion of innovation model: Relative Advantage, Ease of Use, and Trialability. The greater the relative advantage, ease of use, and trialability of the PHR, the more patients value the PHR for communicating with their doctor’s office. The fourth domain from the diffusion of innovation model, observability, did not emerge as a significant predictor of the value of the PHR. The remaining three significant predictors are: Privacy/Security, Computer Use, and Age. More positive perceptions of privacy and security of information in the PHR are associated with greater perceived value of the PHR. The use of a computer is associated with a greater perceived value of the PHR. Finally, age has a small but positive effect on perceived value of the PHR.

**Table 4:** Results of multiple regression with the value of PHR for communicating with the doctor’s office as the dependent variable.

Model^a^	Unstandardized coefficients		Standardized coefficients	*t*	Sig.
	B	Std. Error	Beta		
Constant	-7.71	1.08		-7.13	.000
User versus Rejecter^b^	-0.32	0.27	-.05	-1.18	.24
User versus Non-Adopter	-0.04	0.30	-.01	-0.12	.90
Rejecter versus Non-Adopter	0.28	0.35	.03	0.81	.42
Age	0.02	0.01	.08	2.19	.03^c^
Gender^d^	0.30	0.21	.05	1.42	.16
Race^d^	0.07	0.32	.01	0.20	.84
Education^e^	-0.06	0.23	-.01	-0.25	.80
Income	-0.02	0.25	-.004	-0.10	.92
Marital status	0.11	0.23	.02	0.50	.62
Rating of overall health	0.07	0.11	.02	0.64	.52
Self-reported comorbidities	0.06	0.06	.04	1.05	.29
Technology use	-0.002	0.06	-.002	-0.04	.97
Computer use	1.22	0.60	.07	2.03	.04^c^
PIIT	0.13	0.12	.04	1.04	.30
Privacy and security	0.40	0.12	.11	3.25	.001^f^
Ease of use	0.49	0.14	.14	3.44	.001^f^
Relative advantage	1.87	0.14	.51	13.07	.000^g^
Observability	0.13	0.10	.04	1.35	.18
Trialability	0.31	0.11	.09	2.76	.006^c^

^a^ *R* ^2^ = 0.51; Adjusted *R* ^2^ = 0.49; *R* ^2^∆ = 0.51; *F*∆ = 29.92; df1 = 18, df2 = 520; Sig. F change = .000.

^b^ The model contains the three pair-wise comparisons for the three groups.

^c^ *P* < .05.

^d^ Gender is coded as Female = 1, Male = 0; Race is coded as Caucasian = 1, Other = 0.

^e^ For definitions of all other variables, refer to Tables 1-3.

^f^ *P* < .01.

^g^ *P* < .001.

## Discussion

### Principal Results

Our overall objective was to apply a theoretical model, the diffusion of innovation model, to the study of PHR adoption and use. We adopted two conceptual approaches to achieving this overall objective. First, we classified patient groups into the categories of innovators, laggards, other users, rejecters, and non-adopters. We did not find differences between innovators, other users, and laggards on factors such as use of technology, and use of and satisfaction with the PHR. Although innovators were younger than other users and the proportion of females was greater for innovators than laggards, we did not find any differences between the three PHR user groups on two important socioeconomic variables identified by Rogers [26] in his diffusion of innovation model: education and income. Our findings differ from Rogers’ [26] propositions that innovators are highly educated and possess substantial financial resources compared to laggards. One possible explanation for this difference is neither technology nor socioeconomic characteristics distinguish among users of a PHR. A tethered-PHR such as the one that is the focus of this study is not associated with a financial cost of adoption on the part of patients as the innovation is provided by their health care organizations. In contrast, many of the innovations that Rogers studied for his diffusion of innovation model were financially costly to adopt and led Rogers to conclude that socioeconomic status and innovativeness go hand in hand. The other potential explanation for our difference from Rogers’ findings has to do with the heuristic definitions we adopted for our three groups. It is possible that there are other classifications of innovators and laggards that exist that might reveal differences in socioeconomic and technological characteristics. For example, laggards in our study had lower personal innovativeness in information technology than innovators. Thus, one area for additional research on this topic could be the classification of patients as innovators and laggards based on new functionality in the PHR that is driven by personal innovativeness in information technology use.

Our study did find systematic differences between those who use a PHR and those who did not adopt a PHR on income, education, technology use and access, and personal innovativeness in information technology. Non-adopters were not only less educated and had lower income but also had lower use of technology in general and less personal innovativeness in information technology. These results are indicative of the presence of the digital divide in PHR that we and others have reported [9,16,17,20]. However, most of the existing studies on the digital divide associated with PHRs have reported adoption and use rates of PHRs or relied on data from surveys of Internet use. We believe that our study is the first to report on the digital divide through a patient survey of PHR users and non-adopters. As far as we are aware, this is also the first empirical study that shows that the digital divide associated with PHR extends to an individual-trait variable such as perceived innovativeness in the domain of information technology. Non-adopters not only have significantly lower use of technology in general and computers in particular, but they also have significantly lower personal innovativeness in information technology (PIIT) compared to users of PHR. Lower PIIT can have antecedent and consequent impacts on perceptions of the PHR [29]. An antecedent impact of lower PIIT is that non-adopters will likely have to rely on more communication channels to learn about a PHR. A consequent impact of lower PIIT is that non-adopters will need to have more positive perceptions with respect to behavioral intentions toward adopting and using a PHR. As a result, non-adopters with lower PIIT face greater challenges in adopting and using a PHR.

Our second approach to applying the diffusion of innovation model to the study of PHRs was to develop and implement a survey on perceptions of using a PHR (or potential use among non-users) using the five domains identified in the model: relative advantage, compatibility, ease of use, observability, and trialability. Our initial factor analysis of the perception items found that compatibility did not emerge as a distinct domain. This finding is similar to that of Moore and Benbasat [27] who found that compatibility merged with the domain of relative advantage in their study on perceptions of use of personal work stations. Rogers [26] has reported that other studies have found that compatibility and relative advantage are not empirically related. There are two potential explanations for this finding. Compatibility is the degree to which an innovation is perceived as consistent with existent values, past experiences, and needs of potential adopters [26]. It is possible that patients do not perceive the compatibility of using a PHR as a domain for consideration given their past experience with using the Internet. That is, the prior use of the Internet gives patients the meaning and congruence for using an Internet-based tool such as the PHR thereby excluding or mitigating compatibility as a perceptual domain. As a result it is other perceived domains—such as ease of use and relative advantage—that dominate the perceptual space of PHR use. On the other hand, it is possible that the items on compatibility that we adopted in our survey need to be modified, or new items on compatibility need to be developed, to better capture perceptions of compatibility of PHR use that are empirically distinct from relative advantage.

Our final factor analysis yielded four domains hypothesized by the diffusion of innovation model. Ease of use and relative advantage emerged as the top two factors in the model. This suggests that PHRs must be perceived as easy to use and must show a perceived relative advantage over existing practices. The finding on ease of use provides empirical support for the importance of usability of PHRs as reported in the literature [2,3,6,8]. The finding on relative advantage contributes to the literature by showing the importance of demonstrating the relative advantage of PHRs over traditional approaches such as calling the office. Our factor on trialability suggests the importance of allowing patients to try the PHR on a trial basis, while observability reflects the importance of patients being able to observe how a PHR can be used. The scales developed from each of the factors also yield findings that are intuitively expected. PHR users perceive greater ease of use and relative advantage than rejecters and non-adopters. In terms of observability and trialability, PHR users and rejecters perceive greater observability and trialability than non-adopters.

Our final analysis used multiple regression with forced entry to predict the perceived value of the PHR for communicating with the doctor’s office from a number of independent variables. In this study, none of the sociodemographic variables except age were significant predictors of patient perceptions of the value of the PHR for communicating with the doctor’s office. We found age to have a small but positive impact on the perceived value of the PHR. While some studies have reported that age-related cognitive limitations pose a barrier to PHR adoption and use [16], others have not found a digital divide with respect to age in PHR use [11]. Our finding is more aligned with the second study by Tang and Lansky [11], which found that one-third of patients in their sixties and a quarter in their seventies had signed up for the PHR and found it to be valuable. Further research is needed on how older patients adopt, use, and value PHRs given that the relationship between age and use and value of the PHR is moderated by several factors such as socioeconomic status, access and use of computers and the Internet, and literacy and numeracy.

Two self-reports capturing health of patients—overall health status and number of comorbid conditions—were not significant predictors of the perceived value of the PHR for communicating with the doctor’s office. This result is contradictory to several studies that have found that expected need of clinical services and presence of chronic/comorbid conditions are associated with PHR adoption and use [9,13,14]. One possible explanation for the difference is that our outcome variable is perceived value of communicating with doctor’s office and not adoption or use. Thus, while expected need or presence of comorbid conditions may predict adoption and use, it may play less of a role in perceived value of the PHR. In the case of patients with chronic conditions, Archer and colleagues [6] have noted “Simply providing online access to medical records is not useful unless the technology is integrated with the patient’s existing health and psychosocial support infrastructure”. Perhaps patients in our study reflected this view in assessing the perceived value of PHRs thereby generating a lack of relationship between health status and perceived value. On the other hand, the lack of a relationship between health status and perceived value may be specific to the patient population in our study. We also relied on self-reports of health status instead of capturing health status through documented comorbidities in the EHR. Additional research is needed to understand the impact of expected need of clinical services and chronic conditions on perceptions of value of the PHR.

Positive perceptions of privacy and security of information in the PHR were positive related to the perceived value of the PHR. While privacy and security may pose a barrier to adoption of PHRs as reported by several researchers [3,4], PHR users may be less concerned about the privacy and security of information in the PHR as found by Hassol and colleagues [8]. A survey of users similarly found that a majority of users were not worried about privacy of information in the PHR [25]. Our finding also suggests that positive framing of the privacy and security of information in PHRs could lead to positive perceptions of the value of the PHR and thereby influence uptake of the PHR as some have suggested [6].

Among the domains of perceptions of use, relative advantage was the strongest predictor of perceived value of the PHR by far followed by ease of use and trialability. Observability did not emerge as a significant predictor. As this is one of the first studies to report such findings, additional research is needed to confirm these findings or suggest alternative findings. Previous analyses of PHRs have identified a set of attributes of the ideal PHR [1,4]: (1) PHRs should be protected, private, and secure; (2) PHRs should be accessible from any place, at any time, and transparent; (3) Ownership of the PHR should lie solely with the customer; (4) The patient should approve storage and use of data; and (5) The data should be in a format that is understandable. Our research, which defines a PHR as an innovation and focuses on perceptions of PHR use, suggests that the following attributes are also important: (1) The PHR must be easy to learn and use; (2) The PHR must provide a relative advantage over traditional approaches such as a phone for contacting the doctor’s office; (3) The PHR must be trialable in that patients must have adequate opportunities to try a PHR; and (4) The PHR must be observable so that patients have an opportunity to observe its features and functionality.

### Concluding Remarks

We conclude that the Rogers model of diffusion of innovations fits well for this innovation and offers insights that are both prescriptive and theoretical in nature with respect to PHR adoption and use. The criteria of meaningful use of EHRs of the HITECH Act that pertain to patient engagement in health care in both Stage 1 [7] as well as subsequent stages place a spotlight on PHR adoption and use given the potential role of the PHR in implementing such criteria. The findings of our study have prescriptive relevance for improving the adoption as well as the use of PHRs so that they can better facilitate the implementation of meaningful use. To improve PHR adoption, especially among rejecters and non-adopters of the PHRs, our study suggests that the most important domains are relative advantage and ease of use of the PHR. While this statement may appear to be fairly straightforward, many PHRs in current use were developed several years ago and it is not clear whether evaluative studies on the ease of use of these PHRs, especially where non-adopters are concerned, have been conducted (at least we have not come across them in the research or practitioner literatures). Additionally, efforts to improve uptake of PHRs among non-adopters could focus on strategies that highlight the relative advantage of a PHR. For example, an intervention to increase the uptake of PHRs could focus on the role of a PHR in avoiding phone tag with a nurse for a prescription refill or the benefits of asynchronous communication offered by the PHR for non-emergent medical questions (eg, patients can email their doctor at their convenience). Practitioners can also address trialability of PHRs among non-adopters, as for example through a computer in a waiting room or through group sessions that educate patients about the use of PHRs. As implementation of meaningful use of EHRs accelerates, it would be worthwhile for practitioners to conduct and share the results of studies that facilitate improvements in domains of perceptions such as ease of use, relative advantage, and trialability of PHRs. In terms of improving the use of PHRs, given that PHR users do not differ with respect to technology and socioeconomic characteristics regardless of their time of adoption, our study suggests that initiatives to improve use of PHR can target all users and do not have to be tailored on whether the patient is an innovator or a laggard. Nonetheless, innovators may be able to play a role as change agents or opinions leaders in the diffusion of PHRs, and this is an important area for further exploration from the standpoint of both research and practice.

Furthermore given that perceived ease of use and relative advantage emerged as important domains in our study, an alternative model for consideration in future research is the Technology Acceptance Model (TAM) proposed by Davis [32,33]. TAM draws upon the work of Fishbein and Ajzen on the relationship between attitudes and behaviors [28]. In TAM, Davis developed a concept called perceived usefulness defined as the “degree to which an individual believes that using a particular system would enhance his or her job performance” [33:477]. Davis found that perceived usefulness was more important than perceived ease of use in predicting usage of technology (our finding in this study is similar in that we found that relative advantage was by far a better predictor of perceived value of the PHR). As Moore and Benbasat [27] point out, the perceived usefulness construct proposed by Davis is similar to the construct of relative advantage in the diffusion of innovation model. The strength of TAM is the inclusion of concepts such as attitudes and behaviors and their linkages to perceptions of use of technology. In a development of TAM, called TAM2, Venkatesh and Davis [34] also incorporate the role of subjective norms in the model. In discussing the use of PHRs for patients with chronic conditions, Winkelman and colleagues [35] reviewed the conceptual relevance of the TAM model for predicting PHR use in such patients but questioned its applicability to the PHR setting given its origins and development for understanding technology use by individuals in the organizational setting.

From a theoretical perspective, our study found evidence to support the application of the diffusion of innovation model to perceptions of the PHR. However, further research is needed in two areas with respect to the model: (1) Are there other definitions of innovators and laggards with respect to PHR use and do these definitions highlight any differences among these groups?; and (2) Is compatibility a perceived domain of PHR use that can be captured separately from relative advantage and ease of use? Another area for potential research is the application of the diffusion of innovation model to standalone PHRs. As reported in the principal results section, contrary to Rogers’ [26] propositions we did not find that innovators are highly educated and possess substantial financial resources compared to laggards. This may be the case with a tethered PHR, which is the focus of this study. But in the case of standalone PHRs, it is possible that innovators differ based on education and financial resources. The factors predicting adoption and use of standalone PHRs may also differ. For example, perceived ease of use may play a more important role in standalone PHRs than relative advantage, as standalone PHRs unlike tethered PHRs do not replace traditional approaches of calling the doctor’s office.

In addition, there is a need to understand the impact of perceptions on such factors as attitudes and behavioral intentions to adopt the PHR as well as the use and sustainability [6] of use of the PHR after adoption. Alternative models to use in such research on PHRs include the TAM model discussed above and the theory of planned behavior developed by Fishbein and Ajzen [28]. In these models, perceptions and attitudes are upstream variables and outcomes such as behaviors, perceived value of the PHR and sustainability are downstream variables. Appendix 2 presents a conceptual framework of different constructs for behavioral research on PHRs. For example, perceptions may influence behavioral intentions to adopt a PHR directly or indirectly through attitudes. Perceived value of the PHR, on the other hand, may be impacted by perceptions directly as this study found or indirectly through behaviors such as the use of specific PHR functionalities. We do not claim that the constructs and relationships shown in Appendix 2 are exhaustive but offer these as a first step in identifying a conceptual framework for behavioral research on PHRs.

Furthermore, the hypothesized relationships in our conceptual framework may not be static but can be dynamic in nature. With respect to perceptions, the lack of stability of the perceived attributes of an innovation was pointed out by Greenhalgh and colleagues [36]. For example, a patient may initially perceive a relative advantage in using the PHR for asynchronous secure communication with the provider. However, if the patient has a negative experience with the use of the PHR for such communication along the way (a lack of response or delayed response from provider), then the perceived value of the PHR for this functionality could drop and the patient may discontinue the use of PHR for such communication. Thus, along with cross-sectional studies, there is a need for longitudinal studies of perceptions of PHRs and the impacts of changes in perceptions on outcomes such as sustainability of PHR use.

Finally, while it is important to understand individual-level factors such as perceptions as examined in our study, a focus on such individual-level factors may lead to what Rogers [26] has called “individual blame” in diffusion of innovation studies. Under the individual blame approach, the patient is blamed for lack of adoption and use of the PHR and inadequate attention is paid to other factors that also impact adoption and use. Here again, Greenhalgh and colleagues [36] offer some important contributions to the literature based on their analysis of innovations in service delivery and organization. Some of the key factors identified by Greenhalgh and colleagues include: (1) psychological antecedents or traits associated with trying and using innovations such as tolerance of ambiguity and motivation; (2) the meaning of the innovation for adopters, which may differ from the meaning of the innovation for the organization implementing the innovation; (3) the context—technological, political, and cultural—within which the innovation is adopted; and (4) organizational-level factors such as structures, processes, and culture. Similar factors have been identified in existing reviews and empirical studies of PHRs including the meaning of the PHR for patients [35], cultural trends in information technology use [2], political and technological factors influencing EHR adoption [6], policies for interoperability of PHRs [4], and organizational strategy for EHR and PHR implementation and use [3,18]. Yet another important factor in counteracting the individual blame approach in PHR adoption and use is the role of provider encouragement and use of the PHR [18]. In field studies that we have conducted on PHR adoption and use, we have repeatedly heard from providers, staff, and patients that if providers are enthusiastic and encourage patients to adopt and use PHRs then patients are more likely to adopt and use PHRs. Thus as the implementation of EHRs and PHRs accelerates under HITECH, we recommend that providers play an active role in the uptake of PHRs and their subsequent use.

### Limitations

This study has several limitations. It is an exploratory study and one of the first to apply the diffusion of innovation model to the empirical study of PHRs. It was conducted in only one system and region, and the results may not be generalizable to other settings. In addition, although as we indicated our response rates were comparable or exceeded many existing studies, the response rates were just over 50%, and non-responders to the survey may have had different perceptions than responders. Non-users and users were not matched also (and you can see this reflected in non-adopters’ greater number of co morbidities). Two of our domains—observability and trialability—had only two items in each domain. Future research should develop and implement more items in each of these domains. Finally, we focused on perceived value of the PHR for communicating with the doctor’s office as our outcome variable. However, as we show in Appendix 2, other relevant outcomes can be examined such as PHR-related behaviors or sustainability of PHR use. Similarly, other predictors may play a role such as attitudes, self-efficacy, and psychological traits. Herein lies the complexity of behavioral social science research related to PHRs. A number of models exist (eg, diffusion of innovations, technology acceptance model, and theory of planned behavior) each providing its own set of predictors and outcomes. At the same time, limited funding and the logistics of research (for example the need to keep surveys short to enhance response rates) force the researcher to select a given model and an associated set of variables as we have done in this study. Hopefully cumulative research over time with different models and frameworks like the one we adopted in this study will build the knowledge base for much needed behavioral research on patient adoption and use of PHRs.

## Multimedia Appendix 1

Survey items on perceptions of PHR use, personal innovativeness in information technology (PIIT), and privacy and security.

## Multimedia Appendix 2

A conceptual framework and hypothesized relationships for behavioral research on PHRs.
