# Supplementary material for: Patient Perceptions of a Personal Health Record: A Test of the Diffusion of Innovation Model
Source: J Med Internet Res. 2012 Nov 5;14(6):e150. doi: 10.2196/jmir.2278 (PMC3517342; doi:10.2196/jmir.2278)
Supplement: Supplementary file 1 [file jmir_v14i6e150_app1.pdf]

Appendix 1. Survey items on perceptions of PHR use, personal innovativeness in information technology (PIIT), and privacy and security[1]

Using Patient Gateway[2] improves the quality of care I receive (RA[3])
Using Patient Gateway gives me greater control over my care (RA)
Using Patient Gateway enables me to contact my doctor's office more quickly (RA)
Using Patient Gateway makes it easier to contact my doctor's office (RA)
The effectiveness of care I receive will not improve by my using Patient Gateway (RA)
Using Patient Gateway will fit in with all aspects of my health care coordination (CO)
Using Patient Gateway fits into my personal and work life (CO)
I think that using Patient Gateway does not fit well with the way I like to receive care (CO)
Using Patient Gateway requires a lot of mental effort (EU)
Using Patient Gateway is frustrating (EU)
Learning to use Patient Gateway was easy for me (EU)
Overall, I believe that Patient Gateway is easy to use (EU)
Using Patient Gateway to communicate with my doctor's office is easy for me (EU)
I did not really have adequate opportunities to try out different features in Patient Gateway (TA)
I tried Patient Gateway on a trial basis to see what it can do for me (TA)
I really did not lose much by trying Patient Gateway, even if I would not have liked it (TA)
I have seen what others can do using Patient Gateway (OB)
I have talked to others about using Patient Gateway (OB)
If I heard about a new information technology, I would look for ways to experiment with it (PIIT)
Among my peers, I am usually the first to try out new information technologies (PIIT)
In general, I am hesitant to try out new information technologies (PIIT)
I like to experiment with new information technologies (PIIT)
The identity of anyone using Patient Gateway would be carefully confirmed by my doctor's office to prevent any unauthorized access or any cases of mistaken identity (Prv/Sec)
The information in my Patient Gateway account will only be seen by my health care providers and not by others who are not authorized to see the information such as employers and insurance companies (Prv/Sec)
My doctor's office will immediately notify me if there was a privacy or security breach related to my information in my Patient Gateway account (Prv/Sec)

1. All items measured on a scale from 1 to 5: 1=Strongly Disagree; 2=Disagree; 3=Neutral; 4=Agree; 5=Strongly Agree

2. Patient Gateway is the name of the PHR that was the focus of this study
3. Indicates the domain of diffusion of innovation into which the item falls: RA = Relative Advantage; CO = Compatibility; EU = Ease of Use; OB = Observability; TA = Trialability

Note: For the non-user survey we used the same items but replaced Patient Gateway with “electronic personal health record” and asked about potential use, as for example: “Using an electronic personal health record will give me greater control over my care” (RA) and Learning to use an electronic personal health record will be easy for me” (EU)
