# Supplementary material for: Patient Perceptions of a Personal Health Record: A Test of the Diffusion of Innovation Model
Source: J Med Internet Res. 2012 Nov 5;14(6):e150. doi: 10.2196/jmir.2278 (PMC3517342; doi:10.2196/jmir.2278)
Supplement: Supplementary file 2 [file jmir_v14i6e150_app2.pdf]

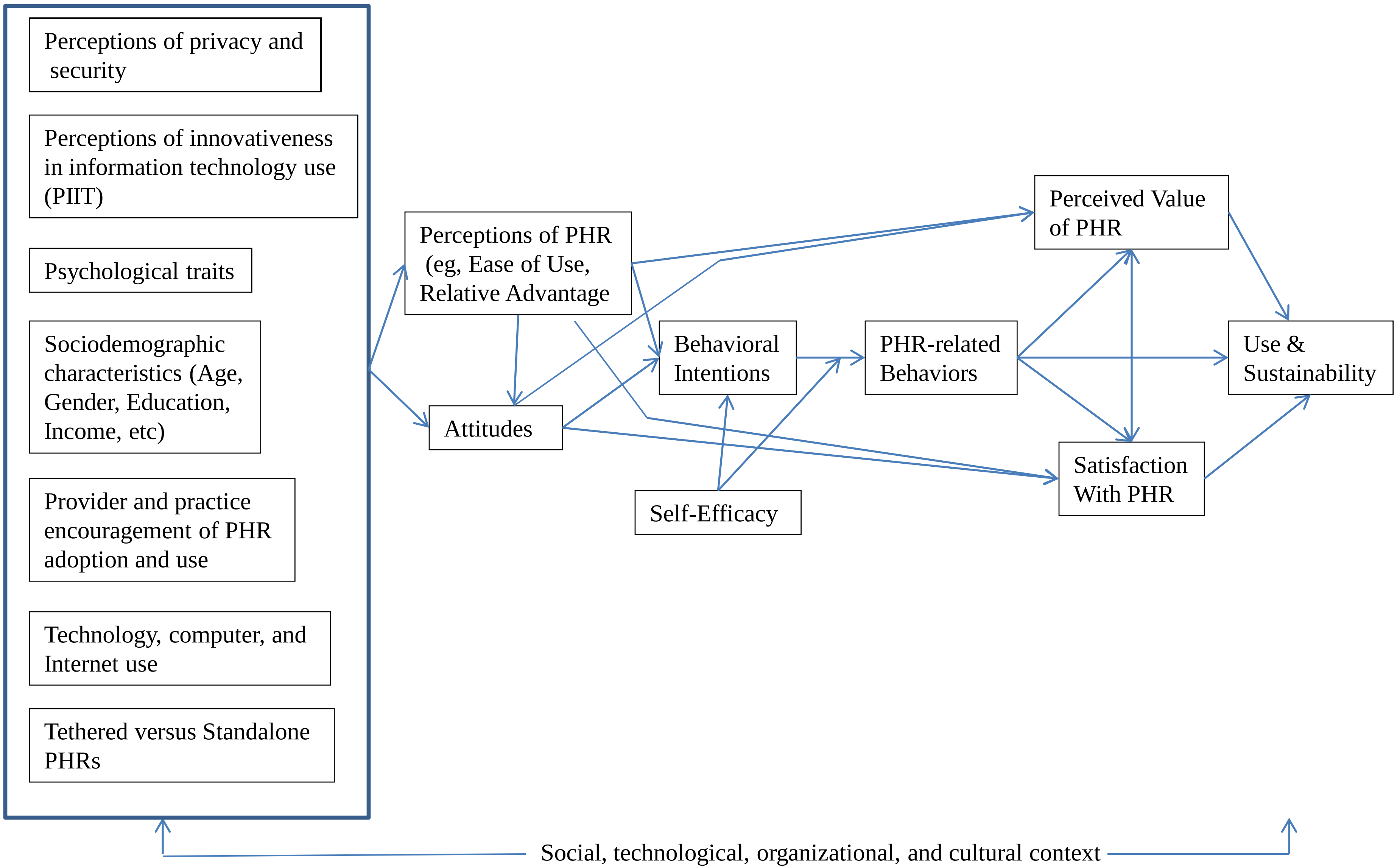

Appendix 2: A conceptual framework and hypothesized relationships for behavioral research on PHRs.
